# The Management of Newly-Diagnosed Large B-cell Lymphoma: A British Society for Haematology Guideline

**DOI:** 10.1111/bjh.19273

**Published:** 2024-01-21

**Authors:** Christopher P Fox, Sridhar Chaganti, Graham McIlroy, Sally F Barrington, Cathy Burton, Kate Cwynarski, Toby A Eyre, Timothy Illidge, Nagesh Kalakonda, Andrea Kuhnl, Pam McKay, Andrew J Davies

**Affiliations:** 1 School of Medicine, University of Nottingham, Nottingham; 2 Centre for Clinical Haematology, University Hospitals Birmingham, Birmingham; 3 Institute of Cancer and Genomic Sciences, University of Birmingham, Birmingham; 4 King’s College London and Guy’s and St Thomas’ PET Centre, School of Biomedical Engineering and Imaging Sciences, King’s College London, London; 5 Department of Haematology, The Leeds Teaching Hospitals, Leeds; 6 Department of Haematology, University College London Hospitals, London; 7 Oxford Cancer and Haematology Centre, Oxford University Hospitals, Oxford; 8 Manchester NIHR Biomedical Research Centre, Division of Cancer Sciences, University of Manchester, Manchester; 9 Department of Molecular and Clinical Cancer Medicine, University of Liverpool, Liverpool; 10 Department of Haematology, King’s College Hospital, London; 11 Department of Haematology, Beatson West of Scotland Cancer Centre, Glasgow; 12 Cancer Sciences Divisions, Centre for Cancer Immunology, University of Southampton, Southampton

## Methodology

This guideline was developed according to the BSH process, as set out on www.b-s-h.org/guidelines. The Grading of Recommendations Assessment, Development and Evaluation (GRADE) nomenclature was used to evaluate levels of evidence and to assess the strength of recommendations (see www.gradeworkinggroup.org).

### Review of the manuscript

The manuscript was reviewed by the BSH Guidelines Committee Haemato-Oncology Taskforce, the Guidelines Committee, and the Haemato-Oncology Sounding Board. It was on the members section of the BSH website for comment. The guideline has also been reviewed by patient representatives nominated by the UK charity Lymphoma Action (www.lymphoma-action.org.uk), this organisation does not necessarily approve or endorse the contents.

## Scope

This British Society of Haematology (BSH) guideline summarises the recommended initial investigation and first-line management of large B-cell lymphoma (LBCL). Primary extra-nodal LBCL is discussed in this guideline, with the exception of lymphoma involving the central nervous system, covered by separate BSH guideline publications.^1, 2^ Post-transplant lymphoproliferative disorders are also covered by a separate guideline.^3^ The investigation and management of primary mediastinal large B-cell lymphoma, mediastinal grey-zone lymphoma, primary cutaneous LBCL, primary effusion lymphoma, plasmablastic lymphoma and Burkitt lymphoma are also beyond the scope of this guideline. Management of relapsed LBCL is covered in a separate guideline.

## Introduction

Large B-cell lymphomas are a biologically heterogenous group of clinically aggressive malignancies arising from mature B lymphocytes. Current classification systems describe a number of LBCL subtypes based on morphological, molecular and clinical characteristics.^4, 5^ Where tumours do not meet the criteria for one of these specific disease entities, they are classified as diffuse large B-cell lymphoma, not otherwise specified (DLBCL, NOS), or high grade B-cell lymphoma, not otherwise specified (HGBCL, NOS). The vast majority of patients will receive systemic chemo-immunotherapy delivered with curative intent. The type of regimen, number of cycles and radiotherapy consolidation are influenced by a range of disease features and patient characteristics. Patients should be actively involved in all aspects of their care, and an understanding of their individual priorities should be established early to ensure person-centred care. Patients can also be signposted towards relevant charities for further information and support in an accessible format.

## Diagnosis and baseline investigations

Sufficient tissue sampling is essential for the accurate classification of LBCL, and excisional biopsy is recommended. However, whilst surgical excision is more likely to yield adequate material,^6^ if it is impractical, entails excessive risk, or confers undue delay, then core biopsy is an acceptable alternative. There is generally no role for fine needle aspiration and it can delay diagnostic tissue biopsy. Expert haematopathology review is essential, employing a full range of phenotypic and molecular investigations. Diagnostic material, framed in a clinical context should be discussed at a multidisciplinary team meeting.

Positron emission tomography (PET) using [^18^F] fluorodeoxyglucose (FDG), combined with low-dose computed tomography (CT) is the recommended imaging modality for staging of LBCL.^7–9^ In most cases, contrast-enhanced full-dose CT does not confer additional value.^10, 11^ PET-CT is more likely than bone marrow biopsy to detect marrow involvement with LBCL, and the presence of non-avid bone marrow disease does not confer a worse prognosis.^12–16^ Bone marrow biopsy may be considered for selected patients in whom a co-existing haematological condition is suspected (for example low-grade lymphoma or myelodysplasia), and where this would inform clinical management, but is otherwise unnecessary. Baseline tumour burden, as assessed by metabolic tumour volume on PET-CT is a promising biomarker.^17, 18^

Where suspected, central nervous system (CNS) involvement should be investigated by contrast-enhanced magnetic resonance imaging (MRI) of the brain and/or spinal cord together with cerebrospinal fluid (CSF) examination (cytology and flow cytometry).^19^ CNS investigations should also be considered for patients at high risk, informed by the CNS-International Prognostic Index (CNS-IPI)^20^ and the number (three or more) and location of extra-nodal sites of disease.^21^

Electrocardiography (ECG) should be performed on all patients prior to chemotherapy. Assessment of left-ventricular function (echocardiography or multi-gated acquisition (MUGA) scan) should be considered for older patients, those with abnormalities on ECG, and patients with a history of cardiovascular disease or risk factors. Patients with abnormal investigations may warrant clinical evaluation by a cardiologist. The significance of serum troponin concentration in this context is not established, and routine testing is not currently recommended.

Serological testing for hepatitis B (to include core antibody), hepatitis C and human immunodeficiency virus (HIV) should be routinely performed prior to starting treatment. Patients with positive serology for hepatitis B should undergo viral DNA quantification and receive prophylactic antiviral therapy and hepatic B virus polymerase chain reaction (HBV PCR) monitoring, both during and after chemo-immunotherapy, as per current guidance.^22^ Referral to a hepatologist or infectious disease physician should be considered. Patients with positive serology for hepatitis C virus (HCV) require HCV RNA quantification and should be urgently referred to a hepatologist or infectious disease physician. Patients testing positive for HIV should be urgently referred for joint care from an HIV specialist at a centre of expertise.^23^ Those with well-controlled HIV (fully-suppressed viral load and CD4 count ≥200x10^6^/L) can be treated on the same protocols as patients who are HIV-negative.

Reproductive counselling should be offered to all age-appropriate patients in whom potentially gonadotoxic therapy is planned. Sperm cryopreservation, ovarian preservation or oocyte harvest should be discussed where relevant. Patients who may be affected by the menopause during or after treatment should be signposted to their general practitioner for counselling, and the relevant investigations and hormonal replacement after completing treatment for lymphoma.

Before starting therapy (including pre-phase treatment), a risk-based prophylaxis and monitoring plan for tumour lysis should be initiated.^24^

### Prognostic assessment

The International Prognostic Index (IPI) should be calculated for all patients.^25^ The National Comprehensive Cancer Network (NCCN)-IPI^26^ may allow better prognostic delineation of both high and low-risk patients.^27^ The stage modified IPI (smIPI) is discriminative in those with localised disease.^28^ Additional information should be incorporated into risk assessment including the presence of bulky disease (≥7.5 cm),^29^ and presence of *MYC* and *BCL2* (with or without *BCL6*) co-translocations.

Both recently revised classifications^4, 5^ describe HGBCL with *MYC* and *BCL2* rearrangements (with or without *BCL6* rearrangement) as an aggressive lymphoma of GCB origin with distinct biology from other LBCLs. Data to support distinct biology in patients with *MYC* and *BCL6* rearrangements are less compelling. In the WHO-HAEM5,^4^ dual *MYC* and *BCL6* rearrangements are now classified either as a subtype of DLBCL, NOS or HGBL, NOS according to their cytomorphological features. The revised ICC^5^ has retained HGBCL-DH-BCL6 as a provisional entity to allow for continued study. *MYC* translocation to an immunoglobulin partner is most strongly associated with inferior overall survival.^30^ Other predictors of poor outcome, for example *TP53* mutations and ‘molecular high-grade’ gene expression signature,^31, 32^ are not yet routinely used in clinical prognostication.

Gene expression profiling distinguishes molecular subtypes of LBCL according to the cell-of-origin (COO) model; a germinal centre B-cell (GCB) pattern is associated with more favourable outcomes than activated B-cell (ABC) disease.^33–35^ Whilst surrogate COO delineation is possible with immunohistochemistry,^36^ and the distinction is retained in current consensus criteria,^4, 5^ it has limited utility in routine diagnostic practice outside clinical trials. More recently, comprehensive genomic approaches have identified a number of LBCL sub-groups but the clinical utility of this approach has not yet been established.^37–39^

Table 1 provides a summary of the investigations to be performed or considered at baseline. Investigations should be coordinated to minimise hospital visits and ensure the timely collation of results. Patients should be contacted early by a keyworker (e.g. a lymphoma clinical nurse specialist), who can help them navigate this process.

### Recommendations

- Perform excision biopsy to provide the optimal material for diagnosis. (1B)
- Consider needle-core biopsy when a surgical approach is either impractical or entails excessive risk or delay. (2B)
- Diagnosis should be made in a reference haematopathology laboratory with access to a full range of phenotypic and molecular investigations. (1A)
- Discuss all diagnoses and treatment plans at a fully constituted haemato-oncology multidisciplinary team (MDT) meeting. (1A)
- All patients should have a full range of baseline blood tests to include serum lactate dehydrogenase (LDH) and full serology for hepatitis B (including core antibody), hepatitis C and HIV. (1A)
- Perform baseline PET-CT for all patients. (1A)
- Consider contrast-enhanced CT of neck, chest, abdomen and pelvis as an alternative if PET-CT is not practicable, or as an additional imaging modality in selected cases. (2B)
- Perform contrast-enhanced MRI of the brain (include spine if clinically indicated) and baseline CSF assessment (to include cytology and immunophenotyping) where there is clinical suspicion of CNS involvement. (1B)
- Consider contrast-enhanced MRI of the brain (include spine if clinically indicated) and baseline CSF assessment (to include cytology and immunophenotyping) for those considered at high risk of CNS involvement. (2B)
- Consider staging bone marrow biopsy only where discordant or alternative bone marrow pathology would influence clinical management. (2B)
- Perform a baseline ECG on all patients. (1B)
- Consider a baseline echocardiogram (or alternative imaging) to assess left ventricular function in older patients and those with relevant risk factors (2B)
- Record Eastern Cooperative Oncology Group (ECOG) performance status, International Prognostic Index (IPI) and CNS-IPI scores for all patients. (1A)
- Perform fluorescence *in situ* hybridization (FISH) for *MYC* rearrangements. (1B)
  ○ If *MYC* is rearranged, evaluate for an immunoglobulin gene partner and perform FISH for *BCL2* and *BCL6* rearrangement. (1B)
- Determine COO in line with current classifications. (2B)
- Gene expression profiling and comprehensive genomics are not currently standard of care.
- Discuss and explain all diagnoses and treatment plans with the patient, and their family or carer if appropriate. Signpost to additional sources of support. (1B)

## Supportive care

Optimising all aspects of supportive care is important to reduce morbidity, particularly for elderly or frail patients. Patients often present with complex needs, and involvement of key members of the wider MDT (e.g. lymphoma clinical nurse specialists, pharmacists, cardio-respiratory specialists, and healthcare of older people liaison teams) can prove invaluable.

The risk of osteoporotic bone fractures is significant in the LBCL patient population.^40^ The 18-month cumulative incidence of frailty-related fractures was 11% in a UK study of LBCL patients ≥70 years.^41^ A predisposing history (osteoporosis, osteopenia, prior fracture and rheumatoid arthritis), bony involvement with lymphoma and receipt of pre-phase corticosteroids were independent risk factors. Baseline osteoporosis risk should be assessed (e.g. FRAX score). Patients receiving steroid therapy have been shown to benefit from vitamin D treatment in other contexts so this may be considered.^42^ Bisphosphonate or similar therapies should be considered in patients at higher risk.^43^

Infection is a common cause of morbidity and mortality in the context of LBCL therapy.^44, 45^ Primary granulocyte colony-stimulating factor (G-CSF) prophylaxis and antimicrobial prophylaxis should be considered for all patients.^46^ Neutropenic prophylaxis with fluoroquinolone may be considered - informed by local microbiological guidance - for example in patients with additional risk factors for infection. Treatment should be delivered in an appropriate clinical setting with adequate staffing, so that immediate complications can be managed according to applicable guidelines.

### Recommendations

- Consider referral to relevant specialities (e.g. cardiology, health care of older people, endocrinology) for medical optimisation prior to and/or during treatment. (2B)
- Clinically assess osteoporosis risk in all patients. (1B)
- Consider vitamin D supplementation and bisphosphonate treatment according to risk profile. Consider seeking advice from an endocrinologist. (2B)
- Offer primary G-CSF prophylaxis to all patients receiving chemo-immunotherapy with curative intent. (1B)
- Consider primary prophylaxis against herpes simplex/zoster (e.g. aciclovir) and *Pneumocystis jirovecii* (e.g. co-trimoxazole). (2B)

## Stage I and II disease

Up to one third of patients with LBCL present with early stage (I/II) disease, some of whom can undergo abbreviated systemic therapy (see below). Bulk has been conventionally defined as a maximal tumour diameter of ≥7.5 cm, although it is recognised that bulk is a continuum.^29^ A number of approaches to clinical management have evolved, although the heterogeneous populations included in the key trials introduces complexity to clinical decision-making; individualised multidisciplinary discussion is required.

A minority of patients with early-stage LBCL will fulfil the eligibility criteria of the randomised phase 3 FLYER trial:^47^ age 18-60 years with an IPI of 0 and non-bulky disease. Such patients should be offered abbreviated chemo-immunotherapy with four cycles of R-CHOP plus two additional rituximab doses, as this was non-inferior to six cycles of R-CHOP (3-year progression-free survival (PFS) 96% vs 94%), with fewer adverse events.

Preliminary data from the randomised phase 3 LYSA LNH 09-1B trial support an interim (i)PET-adapted approach.^48^ Eligibility criteria were broader than for the FLYER study: ages 18-80, age-adjusted (aa)IPI=0, bulky disease permitted. Patients were randomly allocated to standard treatment with six cycles of R-CHOP, or a PET-adapted experimental arm where patients in complete metabolic response (CMR) after two cycles received two further cycles of R-CHOP. The PET-adapted approach was non-inferior (3-year PFS 92% vs 89%). Further support for iPET-adapted therapy is provided by the prospective S1001 trial,^49^ which showed excellent outcomes (5-year PFS 89% and overall survival (OS) 91%) after four cycles of R-CHOP in the 89% of patients who were in CMR on iPET3. A retrospective analysis of patients with early-stage disease also described high rates of long-term disease control (5-year PFS 88% and OS 90%) with iPET3-directed abbreviated R-CHOP alone.^50^ However, the less favourable outcomes for iPET3-positive patients, including those treated with combined modality therapy, underscore that the optimal approach for these patients is not yet clear.

Where a risk-adapted abbreviated chemo-immunotherapy strategy is not planned, combined modality treatment with abbreviated chemo-immunotherapy plus radiotherapy is generally recommended.^28, 51^ Long-term follow up data from these and other studies have emphasised the risk of late relapses after first-line treatment for early-stage disease. Notably, outcomes appear to be similar amongst patients treated with combined modality and standard chemo-immunotherapy approaches.^52^ When offered, involved site radiation therapy (ISRT) should be delivered according to internationally agreed guidelines.^53, 54^

A proportion of patients with stage I or II disease show adverse risk features, including a high IPI or bulky disease. This group was recognised in a phase 3 randomised trial, in which all patients with a high stage-modified IPI received 6 cycles of R-CHOP, however an incremental benefit from radiotherapy was not demonstrated.^55^ Additional data on the benefit of radiotherapy after full-course chemo-immunotherapy are mixed and limited to retrospective studies. Individual factors must be considered, including distribution of disease at baseline, quality of treatment response, and the relative risks and benefits of radiotherapy consolidation.^54^ The phase 3 POLARIX trial comparing RCHP-polatuzumab vedotin with R-CHOP (discussed in more detail in the Advanced Stage section of this guideline) included 11% of patients with stage I/II disease and IPI ≥2.^56^ The relatively small number of patients limits further interpretation of the early-stage subgroup. However their inclusion within the intention-to-treat POLARIX population means RCHP-polatuzumab vedotin is an option for those with high IPI.

In early-stage disease, double-hit (*MYC* and *BCL2* or *BCL6* rearrangement) and high-risk COO status were not associated with poorer PFS or OS.^57–59^

### Recommendations

- Consider well-designed clinical trials as an option for all patients (2A)
- Where more than one treatment approach is suitable, treatment decisions should be guided by the MDT and in accordance with patient preferences. (2A)

*For patients 18-60 years with stage I/II, aaIPI 0, without bulky disease: offer abbreviated chemo-immunotherapy*

- Offer 4 cycles of R-CHOP plus 2 additional infusions of rituximab. (1A)
- Radiotherapy is not required if full dose intensity is delivered. (1A)

*For patients 61-80 years with stage I/II, aaIPI 0, without bulky disease: consider a PET-adapted approach*

- Perform iPET2 after 2 initial cycles of R-CHOP
  ○ If PET2 CMR (Deauville score (DS) 1-3), complete treatment with a further 2 cycles of R-CHOP. (1A)
  ○ If PET2 not CMR (DS 4 or 5), deliver 4 further cycles of R-CHOP followed by radiotherapy consolidation (ISRT 30 Gy in 15 fractions). (1B)

*For other patients <80 years with stage I/II disease and IPI 0-1*

- Consider combined modality therapy with 3-4 cycles of R-CHOP followed by radiotherapy consolidation (ISRT 30 Gy in 15 fractions). (2B)
- Consider 6 cycles of R-CHOP without radiotherapy where the risks of radiation are considered to be greater than 2-3 further cycles of R-CHOP. (2B)

*For other patients <80 years with stage I/II disease and IPI ≥2*

- Offer 6 cycles of RCHP-polatuzumab vedotin or R-CHOP followed by end-of-treatment PET-CT. (1B)
- Consider radiotherapy consolidation according to baseline disease bulk and distribution, treatment response, and risks of radiation (ISRT 30 Gy in 15 fractions). (2B)

*Additional considerations for radiotherapy consolidation*

- Consider radiotherapy consolidation, following response to chemo-immunotherapy (including where an iPET2 CMR has been demonstrated), for patients with stage I/II disease patients in the following scenarios, unless the toxicities of radiotherapy are considered to outweigh potential benefits:
  ○ Patients receiving less than full-dose intensity of chemo-immunotherapy (including those receiving RminiCHOP or less intensive rituximab-chemotherapy combinations). (2B)
  ○ Patients with extra-nodal involvement (for specific primary extra-nodal sites refer to next section). (2B)
  ○ Patients with bulky disease (≥7.5 cm diameter). (2B)

## Primary extra-nodal LBCL

Management of primary extra-nodal LBCL is an area of uncertainty. Patients with specific extra-nodal localisations and primary extra-nodal disease (compared with extra-nodal extension of predominantly nodal disease) are not individually well-represented in clinical trials of early-stage disease. Involvement of specific extra-nodal sites may have distinct therapeutic implications. Some extra-nodal subtypes are associated with inferior prognosis, and treatment usually follows an advanced stage LBCL approach. For specific sub-types, there are limited data for applying a combined modality approach with abbreviated chemo-immunotherapy. Most patients with primary extra-nodal LBCL have a low IPI. However those with IPI 2-5 were eligible for the POLARIX trial, and RCHP-polatuzumab vedotin is an option in this group. The role of CNS prophylaxis, particularly in patients with primary breast LBCL, remains an area of uncertainty.

Testicular involvement by LBCL is associated with an inferior clinical outcome compared with other subtypes of extra-nodal LBCL, in particular with a higher risk of CNS relapse. Management of this LBCL subtype is often based on the protocol used in the phase II IELSG10 study which involved six to eight cycles of R-CHOP, four doses of intrathecal methotrexate and radiotherapy to the contralateral testis and regional lymph nodes (where involved). Outcomes in the 53 patients enrolled were favourable compared with historical controls, with 5-year PFS and OS of 74% and 85% respectively, and cumulative incidence of CNS relapse of 6%.^60^ The subsequent IELSG30 trial employed the same chemotherapy/radiotherapy protocol but additionally incorporated 4 doses of intrathecal liposomal cytarabine and 2 cycles of intravenous methotrexate (1.5 g/m^2^) following completion of R-CHOP chemotherapy. Recently presented data from this study (n=54) reported no CNS relapses during the median 6 years of follow-up, although late extra-nodal relapses were observed. A PFS of 88% at median follow up of 5 years was reported.^61^

Data to inform treatment of primary breast LBCL is largely retrospective, but several studies have reported high rates of relapse both in the ipsilateral and contralateral breast and in the CNS.^62, 63^ As a result, CNS prophylaxis and consolidation radiotherapy are often considered. Six cycles of chemo-immunotherapy are typically delivered.^62^

Patients with gastric LBCL are generally treated with 6 cycles of R-CHOP.^64^ Combined modality treatment with 3-4 cycles of CHOP followed by radiotherapy has previously shown activity.^65^ However, there is very limited evidence for a role of radiotherapy after chemo-immunotherapy, and irradiation of the stomach is often avoided. Surgery is generally avoided unless required for complications such as perforation, obstruction or bleeding. The absolute risk of gastric perforation is low; elective hospital admissions are usually not required.^66^

Intravascular LBCL (IVL) is rare and characterised by neoplastic B cells within the lumen of blood vessels. These lymphomas more commonly affect older patients and often involve the CNS (approximately 30% of patients), lungs and skin. Prognosis is generally poor although patients with isolated skin lesions appear to experience more favourable outcomes (3-year OS: 56% versus 22%).^67^ Rituximab has had a significant impact upon outcomes in this disease although it may be associated with severe infusion related reactions.^68, 69^ This may be mitigated during initial induction by delaying rituximab until 2-3 days after the first dose of chemotherapy. Seeking to mitigate the high risk of CNS involvement, an early-phase trial incorporating intrathecal and high-dose intravenous methotrexate into an R-CHOP backbone reported a 2-year PFS of 76%.^70^

Primary cutaneous LBCL, leg type typically presents in older people with rapidly growing tumours on one or both legs, but 10-15% of cases arise at other sites and dissemination to extracutaneous sites is common. *MYD88* L265P mutations are found in up to 60% of cases. Survival outcomes were historically poor but data from the recent SEER study suggest improved survival in the rituximab era with 5-year OS of 59%.^71^ Tolerance of systemic therapy is often limited in this older population.

Primary bone LBCL is rare, and bone disease is more commonly seen as a secondary site of widespread stage IV disease.^72^ A pooled analysis of nine prospective trials of newly-diagnosed DLBCL, which identified 1.4% of patients as having primary bone disease, suggested a beneficial effect of radiotherapy to involved sites.^73^

In one retrospective study, radiotherapy was associated with improved outcomes in patients with early-stage extra-nodal LBCL, although the benefit is less clear in patients who are PET-negative at end of treatment.^74^ Consolidation radiotherapy approaches for primary extra-nodal disease may involve irradiation of the whole affected organ. The definition of the clinical target volume for irradiation should be informed by the site of disease, involved tissue volume, and the potential for microscopic residual disease.^75^

### Recommendations

- Consider well-designed clinical trials as an option for all patients (2A)
- For some patients with early-stage primary extra-nodal LBCL, the guidance for early-stage disease can be followed with exception of the primary disease locations listed below, which are generally treated with 6 cycles of chemo-immunotherapy and site-specific recommendations. (2B)
- Consider RCHP-polatuzumab vedotin for patients fit for full-dose chemotherapy with ECOG PS 0-2 and IPI 2-5. (2B)
- CNS prophylaxis is recommended in line with the current BSH good practice paper (GPP) on CNS prophylaxis.^76^ (2B)

*Testicular*

- Offer 6 cycles of R-CHOP. (1B)
- Offer CNS prophylaxis guided by the current BSH GPP. (1B)
- Offer contralateral testicular radiotherapy following completion of systemic therapy. (1B)

*Breast*

- Offer 6 cycles of R-CHOP. (1B)
- Consider consolidation radiotherapy to the involved breast to reduce risk of local recurrence in bulky or localised tumours. (2C)
- Consider CNS prophylaxis as per BSH GPP. (2B)

*Gastric*

- Offer 6 cycles of R-CHOP. (1B)
- Where *H. pylori* is detected, offer eradication therapy as per current guidance. (1A)

*Intravascular LBCL*

- Perform contrast-enhanced MRI of the brain (include spine if clinically indicated) and baseline CSF assessment (to include cytology and immunophenotyping) as baseline screening for CNS disease. (1B)
- Offer 6 cycles of R-CHOP. (1B)
  ○ Consider delaying the first rituximab infusion by ≥48 hours after chemotherapy administration to mitigate against the risk of a severe infusion reaction in cycle 1. (2B)
- Offer CNS prophylaxis as per BSH GPP for those with no evidence of CNS disease at baseline. (1B)
- Offer intensive CNS-directed secondary CNS lymphoma protocols for those with evidence of CNS disease at baseline. (1B)

*Leg-type cutaneous LBCL*

- Offer 6 cycles of R-CHOP. (1B)
- Offer consolidation radiotherapy to areas of previous bulky disease or localised, non-bulky tumours after completion of chemotherapy. (1C)

*Bone*

- Offer 6 cycles of R-CHOP. (1B)
- Consider consolidation radiotherapy to reduce risk of local recurrence in bulky or localised tumours. (2B)

## Advanced stage disease

For over two decades, six cycles of R-CHOP delivered on a 21 day cycle was a well-established standard of care for the majority of patients with newly diagnosed advanced stage LBCL.^77–81^ However, data from the phase 3 POLARIX randomised controlled trial (RCT), in which the CD79b-directed antibody-drug conjugate (polatuzumab vedotin) was incorporated into the RCHP regimen in place of vincristine, reported an improved 2 year PFS (hazard ratio (HR) 0.73, p=0.02; 2-year PFS 76.7% vs 70.2%) for the experimental arm (RCHP-polatuzumab vedotin). Eligible patients had newly diagnosed, *de novo* LBCL with performance status of 0-2, IPI score of 2-5 and suitable for 6 cycles of full-dose R-CHOP.^56^ To-date, no difference in OS is evident. Toxicity profiles of the two regimens were very similar although higher rates of diarrhoea and febrile neutropenia were observed in the experimental arm. Post-hoc analyses from subgroups within POLARIX suggest a greater benefit from RCHP-polatuzumab vedotin amongst patients with IPI 3-5 or ABC COO by GEP. However, the POLARIX trial was not powered to provide evidence for specific subgroups and no sensitivity analyses have been presented. It is therefore not possible to draw firm conclusions about relative treatment efficacy in different sub-groups.

Long-term follow-up data from the REMoDL-B trial (investigating the addition of bortezomib to R-CHOP) recently reported significant differences in both PFS for patients with a molecular high-grade (MHG) gene expression profile (55% vs 29% 5-year PFS; HR 0.46, p=0.011) and also OS for patients with an ABC profile (80% vs 67% 5-year OS; HR 0.58, p=0.032) favouring the bortezomib arm.^32, 82^ COO was determined by transcription profiling, which is not yet available in routine clinical practice. Both treatment arms in REMoDL-B provided six cycles of chemo-immunotherapy, without two additional doses of rituximab, providing a rationale for this treatment approach that is now standard UK practice. Six cycles of chemo-immunotherapy is also an accepted standard in international phase 3 trials of first-line treatment for LBCL. Both treatment arms in REMoDL-B included six cycles of chemo-immunotherapy, without two additional doses of rituximab, providing a rationale for this treatment approach that is now standard UK practice. Six cycles of chemo-immunotherapy is also accepted as standard treatment in international phase 3 trials of first-line treatment for LBCL.^83, 84^

Two randomised phase 3 trials have compared the dose-intensive ACVBP regimen with CHOP, first without and subsequently with concurrent rituximab.^85, 86^ Whilst improvements in event-free survival (EFS) and OS were seen with intensified treatment, the substantially higher rates of toxicity have limited its widespread adoption. An advantage of adding etoposide to R-CHOP had not been demonstrated in a RCT. Delivery of R-CHOP on a 14-day schedule is not superior to a 21-day schedule, but offers a shorter time on treatment.^81^ Consolidation with high-dose chemotherapy (HDT) and autologous stem cell transplantation (ASCT) may confer improvement in EFS for some patients, however the lack of OS benefit means ASCT is reserved as consolidation for patients in second remission.^87–89^

For patients with high-risk disease (IPI 3-5), data from an NCRI single-arm phase 2 trial investigating the intensive R-CODOX-M/R-IVAC regimen described encouraging outcomes for an adverse-risk group: 2-year PFS (68%) and OS (76%).^90^ However, higher rates of treatment-related morbidity and mortality, particularly in patients >50 years or with impaired performance status, should be noted. The dose-intensive, infusional regimen DA-EPOCH-R was compared with R-CHOP in the randomised phase 3 Alliance/CALGB 50303 trial, but no difference in 2-year survival outcomes were observed and the toxicity profile of DA-EPOCH-R was unfavourable.^91^

The prospective PETAL trial investigated dose-intensification of conventional chemotherapy for patients with a positive early interim PET after two cycles of R-CHOP.^92^ There was no observed benefit for dose-intensification over continued treatment with R-CHOP (2-year PFS 41% vs 56%, OS 47% vs 65%), and toxicity was significantly greater in the intensified treatment arm. Non-randomised evidence from the GAINED trial described favourable outcomes for a group of iPET2+ patients who subsequently received HDT-ASCT consolidation. However, this only applied to those who converted to CMR on iPET4 after two further R-CHOP cycles. Although encouraging, these data are not readily applicable to clinical practice given the uncertainties associated with lack of randomisation.^93^

There is no uniform approach towards consolidation radiotherapy (for example to sites of initial disease bulk, extra-nodal sites) in the context of advanced stage disease after full course chemo-immunotherapy. A retrospective analysis of patients with advanced-stage LBCL treated with 6-8 cycles of R-CHOP described the potential role of end-of-treatment PET in guiding consolidation radiotherapy decisions.^94^ In this population-based study, 72% of patients achieved CMR and experienced a 3-year time-to-progression (TTP) of 83% and OS of 87%; baseline bulky disease (≥10 cm) did not appear to impact outcomes in the CMR group. Of the patients not in CMR after initial chemo-immunotherapy, 53% subsequently received radiotherapy, and their outcomes were similar to the PET-negative cohort (3-year TTP 76%, OS 80%). The poorest outcomes were seen in those not in CMR who did not receive radiotherapy. It should be noted however, that 30% of patients not in CMR who did not receive radiotherapy did not experience relapse, reflecting a clinically important false-positivity rate in end-of-treatment scan (see also section on end-of-treatment response). Treatment decisions should be individualised, taking into account potential benefits and anticipated toxicity of the target field, and the potential for subsequent effective therapies in the event of disease relapse.^54^

### Recommendations

- Consider well-designed clinical trials as an option for all patients. (2A)
- Offer 6 cycles of RCHP-polatuzumab vedotin as first-line treatment for patients with de novo LBCL, fit for full dose chemotherapy, with an ECOG PS ≤2 and an IPI score of 2-5. (1A)
  ○ R-CHOP can also be considered as an option for this group. (2A)
- Offer 6 cycles of R-CHOP as first-line treatment for patients with stage III/IV disease and an IPI score of 1. (1A)
- Consider a corticosteroid pre-phase and/or dose-attenuation (e.g. 50%) of cytotoxic agents for the first treatment cycle for patients with impaired PS and/or significant physiological compromise due to advanced stage disease. (2B)
- Consider more dose-intensive regimens such as R-CODOX-M/R-IVAC for younger patients (e.g. ≤50 years of age) with good performance status with IPI ≥3, particularly for those considered at high-risk of CNS relapse. (2B)
- Consider CNS prophylaxis for carefully selected high-risk patients in line with current BSH GPP.^76^ (2B)
- Consider radiotherapy consolidation for bulky disease on a patient-by-patient basis. (2B)
- There is no accepted standard of care for patients with *high-grade B-cell lymphoma with MYC and BCL2 and/or BCL6 rearrangements*. Treatment options include:
  ○ RCHP-polatuzumab vedotin for patients with an IPI score of 2-5. (2B)
  ○ 6 cycles of R-CHOP-21 or R-CHOP-14. (2B)
  ○ For selected younger patients (where *MYC* is translocated to an Ig partner gene and co-exists with a *BCL2* rearrangement), more intensive regimens such as DA-EPOCH-R or R-CODOX-M/R-IVAC may be considered. (2B)
- HDT-ASCT consolidation is *not* recommended as first-line therapy. (1B)

## Older patients and those with co-morbid conditions

### Older patients

For older, frailer patients or those with co-morbid conditions that preclude the delivery of full dose R-CHOP, survival outcomes remain unsatisfactory. Historically, patients enrolled in LYSA and RICOVER-60 trials were considered ‘older’ if >60 years.^77, 80^ This definition has since evolved considerably; the recent SENIOR trial exclusively enrolled patients ≥80 years.^95^

Numerous scoring systems have evaluated patient factors (age, performance status, nutritional status, social support, polypharmacy, activities of daily living (ADLs), comorbidities etc.) but are typically cumbersome, impractical, and rarely performed.^96, 97^ A recent simplified geriatric assessment (sGA) has been validated in 1163 patients classifying patients >64 years as fit (55%), unfit (28%) or frail (18%).^98^ The elderly prognostic index (EPI) integrated the sGA score, International Prognostic Index, and haemoglobin levels to define low (24%), intermediate (48%), and high (29%) risk groups with divergent 3-year OS of 87%, 69% and 42% respectively.^98^ However, the utility of such systems to better inform therapeutic decisions beyond standard clinical assessment remains to be established. In the UK, half of patients presenting with LBCL are over the age of 70 years, with a variable burden of co-morbid conditions.^99^

The evidence base supporting the use of attenuated R-CHOP is primarily limited to single-arm trials or retrospective series, and dosing decisions must weigh up the risk of treatment-related toxicities versus reductions in dose intensity.^100^ In patients <80 years, evidence suggests that retaining full dose intensity of R-CHOP is important for improving survival outcomes.^101^ However, in those ≥80 years studies suggest equivalent survival outcomes between full dose R-CHOP and ‘attenuated’ or R-miniCHOP.^100, 102–105^ Two large phase II trials support the curative potential of ‘mini’ CHOP (doxorubicin (25 mg/m^2^), cyclophosphamide (400 mg/m^2^) and vincristine (1 mg capped-dose)) plus anti-CD20 monoclonal antibody treatment.^106, 107^ Patients ≥80 years treated with the R-miniCHOP regimen experienced a 2-year OS of 59%. In the recent phase III SENIOR trial, the 2-year OS of the R-miniCHOP arm was 67%. Treatment-related mortality was considerably lower with the use of pre-phase steroids and vincristine; likely achieved through improved PS and a lower risk of clinically significant early toxicities.^95, 107^ A recent systematic review supports the notion that R-miniCHOP should be standard of care in suitable patients ≥80 years.^101^

For patients receiving attenuated R-CHOP for limited stage LBCL, there is no firm evidence to guide the optimal dose or number of treatment cycles. Radiotherapy can be an important adjunctive treatment in this age group where long-term risks of radiation may be less relevant.

Holistic, multi-disciplinary care is essential for all patients with LBCL, and is especially important for meeting the complex needs of elderly or frail patients. Timely and regular discussions with patients, carers and family members to understand their priorities, and early involvement of palliative care services, are essential. Many sites are developing frailty services for integrated assessment and management.

A palliative approach that focuses on quality of life and symptom control may be more suitable for very frail or elderly patients. This may include low-intensity cytotoxic regimens, rituximab monotherapy, corticosteroids, palliative radiotherapy, or best supportive care alone for selected patients. There is a clear need for greater clinical research focus, including interventional trials, for the frail and elderly groups.

### Anthracycline-unsuitable patients

A number of non-anthracycline regimens have been developed for patients with significant cardiac history or impaired left ventricular ejection fraction precluding anthracycline use. It should be recognised that intra- and inter-study heterogeneity exists with regard to definitions of cardiac morbidity and ‘unsuitability’ for anthracyclines. A single-arm phase II study of RCVP plus gemcitabine (RGCVP) was conducted in 62 patients.^108^ Two-year PFS and OS were 50% and 56% respectively. R-Gem-Ox-14 (rituximab-gemcitabine-oxaliplatin) was studied in 61 patients (median 75 years) with a 3-year PFS of 49%.^109^ A retrospective series of anthracycline-unsuitable patients supports the substitution of etoposide in place of doxorubicin (50 mg/m^2^ intravenously on day 1, and 100 mg/m^2^ orally on days 2-3) conferring a 4-year OS of 49%.^110–112^ A retrospective Danish population-based study focused on the very elderly (>85 years) suggested comparable overall survival using non-anthracycline regimens (CVP+/-R or CEOP+/-R) as compared with R-CHOP/RCHOEP.^103^ Studies evaluating alternative anthracyclines and bendamustine yielded disappointing results.^113, 114^

### Recommendations

- Consider using a validated frailty index such as the EPI, if practicable, to help inform risks and benefits of therapy (2B)
- Offer a corticosteroid pre-phase (e.g. 1 mg/kg of oral prednisolone) in patients whose performance status is adversely affected by LBCL disease burden. (1B)
  ○ Consider adding vincristine to corticosteroid pre-phase treatment (e.g. a single intravenous 1 mg dose). (2B)
- Offer R-miniCHOP (50% dosing of cyclophosphamide (400 mg/m^2^), doxorubicin (25 mg/m^2^) and vincristine (1 mg)) to patients ≥80 years. (1A)
- Consider dose-attenuation (e.g. 50% or 75%) of the chemotherapy components of R-CHOP in patients <80 years with clinically significant non-cardiac co-morbidities or impaired performance status. (2B)
- Offer a non-anthracycline-based regimen for patients with cardiac co-morbidities unsuitable for anthracyclines; options include RGCVP and RCEOP. (1B)
- Consider palliative approaches (low-intensity cytotoxic regimens, rituximab monotherapy, corticosteroids, palliative radiotherapy, or best supportive care alone) for very frail patients. (2B)

## End-of-treatment response assessment and follow-up

Prospective data from the randomised phase 3 GOYA trial demonstrated that CMR on end-of-treatment PET is highly predictive of favourable long-term outcomes, independent of baseline IPI.^115^ Retrospective studies have similarly demonstrated the prognostic significance of CMR when adjusting for baseline IPI and COO classification.^116, 117^ In one study, 30% of patients not in CMR and who did not receive further treatment nevertheless did not show disease progression, demonstrating a false-positive rate of PET.^94^ Where feasible, biopsy should be performed where there is clinical or radiological suspicion of residual lymphoma, taking into account the clinical context and options for second-line treatment.

The negative predictive value for interim PET is approximately 80%.^118, 119^ A number of studies have scanned patients with newly diagnosed DLBCL at interim and end-of-treatment.^120–123^ The percentage of patients with ‘negative’ iPET and ‘positive’ end-of-treatment PET scans ranged from 0-5%. Interim PET-negative rates range from 50-88%, with a CMR rate of 63% in a prospective blinded study of patients receiving R-CHOP for newly diagnosed DLBCL.^124, 125^ Overall, the majority of iPET scans are negative, and the very low rate of end-of-treatment PET-positivity in this group justifies the omission of the later scan, avoiding the additional radiation dose and cost.

The majority of LBCL relapses occur within the first two years after diagnosis. However, late relapses do occur, including in patients with early-stage disease.^52^ The clinical value to individual patients for routine follow-up within haemato-oncology services beyond two years is not clear. The timing and frequency for follow-up should be informed by the characteristics of the disease, the treatment received and be aligned with the patient’s preferences. It is important to recognise late complications of LBCL therapy, including cardiac disease, bone health, early menopause, neurocognitive effects, and reduced psychological wellbeing.^126, 127^ Patient-initiated follow-up may represent an attractive option for selected patients, although evidence supporting its effectiveness remains limited to date. However, it is clear that patient access to advice and support from haemato-oncology services following completion of therapy is valuable.^128^ The patient’s preferences should inform the mode of follow-up that is offered.

### Recommendations

- Where interim imaging is planned, consider performing a PET-CT following 2 cycles of chemo-immunotherapy (iPET2) for an early assessment of response quality. (2B)
  ○ A change of treatment should *only* be considered for those with no response or progressive disease on iPET2. (1B)
  ○ Patients with early stage LBCL on a PET-adapted protocol should follow the appropriate management plan recommended at the outset. (1A)
- For patients with a complete metabolic response on iPET2, a contrast-enhanced CT scan is usually sufficient as end-of-treatment imaging. (2B)
- For patients without a complete metabolic response on iPET2 (or for patients who have not undergone an iPET2), an end-of-treatment PET-CT scan should be performed. (1B)
- End of treatment response should be assessed 3-6 weeks after the last dose of antibody. (1A)
- End-of-treatment imaging should be performed and reviewed in a timely manner, prior to consolidation radiotherapy or high-dose methotrexate, where planned. (1B)
- Consolidation radiotherapy should commence 6-8 weeks after completion of primary chemo-immunotherapy. (1B)
- After completion of radiotherapy consolidation, re-imaging should be performed at 12 weeks. (1B)
- Metabolic response should be ascertained using the Deauville criteria; DS 1-3 is regarded as CMR. (1A)
- The positive-predictive value of non-CMR is variable; biopsy is therefore strongly recommended prior to second line treatment. (1A)
- For patients with residual foci of FDG-uptake, review imaging in an MDT meeting to assess suspicion of residual disease and amenability to biopsy:
  ○ Offer biopsy of FDG-avid lesions wherever feasible. (1A)
  ○ Offer a repeat PET-CT at an 8-12-week interval, where tissue biopsy is not possible and there is uncertainty regarding imaging findings. (1B)
  ○ Consider ISRT to single FDG-avid lesions if considered suspicious of residual disease but where tissue biopsy is not feasible. Such decisions require careful patient counselling and close consultation with a radiation oncologist. (2B)

## Figures and Tables

**Table 1:** Baseline investigations required, or to be considered, for initial assessment of patients with large B-cell lymphoma.

Required investigations	Investigations to consider if indicated
**Diagnostic biopsy** Excisional biopsy preferred Core biopsy acceptable	Bone marrow biopsy
**Molecular testing** FISH for *MYC* translocation and Ig partner FISH for *BCL2* and *BCL6* translocations if MYC rearranged	
**Baseline blood tests** Full blood count Blood film Renal function and electrolytes Liver function Bone profile LDH Uric acid Immunoglobulins Virology (HBV (including core antibody), HCV, HIV) Blood grouping and antibody screen	Vitamin D
**Imaging** PET-CT	Contrast-enhanced CT
**Central nervous system assessment**	Contrast-enhanced MRI brain and spine CSF flow cytometry
**Cardiac assessment** ECG	Echocardiogram MUGA
